# Palaeoecology of *Voulteryon parvulus* (Eucrustacea, Polychelida) from the Middle Jurassic of La Voulte-sur-Rhône Fossil-Lagerstätte (France)

**DOI:** 10.1038/s41598-019-41834-6

**Published:** 2019-03-29

**Authors:** Denis Audo, Ninon Robin, Javier Luque, Michal Krobicki, Joachim T. Haug, Carolin Haug, Clément Jauvion, Sylvain Charbonnier

**Affiliations:** 1 Yunnan Key Laboratory for Palaeobiology, Yunnan University, North Cuihu road 2#, 650091 Kunming, China; 2 MEC International Joint Laboratory for Palaeobiology and Palaeoenvironment, Yunnan University, Kunming, China; 3 Centre de Recherche en Paléontologie - Paris (UMR 7207), CNRS-MNHN-Sorbonne Université, Muséum national d’Histoire naturelle, Département Origines & Evolution, (CP 38), 8, rue Buffon, F-75005 Paris, France; 4 Department of Geology and Geophysics, Yale University, New Haven, CT 06520-8109 USA; 5 Department of Biological Sciences, University of Alberta, Edmonton, AB T6G 2E9 Canada; 6 Department of Geology, Geophysics and Environmental Protection, AGH University of Science and Technology, Mickiewicza 30, 30-059 Kraków, Poland; 7 LMU Munich, Department of Biology II and GeoBio-Center, Großhaderner Str. 2, 82152 Martinsried-Planegg, Germany; 8 Institut de Minéralogie, de Physique des Matériaux et de Cosmochimie (IMPMC, UMR 7590), Muséum National d’Histoire Naturelle, Sorbonne Université, CNRS, IRD, 57 rue Cuvier, F-75005 Paris, France

## Abstract

Exceptional and extremely rare preservation of soft parts, eyes, or *syn-vivo* associations provide crucial palaeoecological information on fossil-rich deposits. Here we present exceptionally preserved specimens of the polychelidan lobster *Voulteryon parvulus*, from the Jurassic of La Voulte-sur-Rhône Fossil-Lagerstätte, France, bearing eyes with hexagonal and square facets, ovaries, and a unique association with epibiont thecideoid brachiopods, giving insights onto the palaeoenvironment of this Lagerstätte. The eyes, mostly covered in hexagonal facets are interpreted as either apposition eyes (poorly adapted to low-light environment) or, less likely, as refractive or parabolic superposition eyes (compatible with dysphotic palaeoenvironments). The interpretation that *V. parvulus* had apposition eyes suggests an allochthonous, shallow water origin. However, the presence of thecideoid brachiopod ectosymbionts on its carapace, usually associated to dim-light paleoenvironments and/or rock crevices, suggests that *V. parvulus* lived in a dim-light setting. This would support the less parsimonious interpretation that *V. parvulus* had superposition eyes. If we accept the hypothesis that *V. parvulus* had apposition eyes, since the La Voulte palaeoenvironment is considered deep water and had a soft substrate, *V. parvulus* could have moved into the La Voulte Lagerstätte setting. If this is the case, La Voulte biota would record a combination of multiple palaeoenvironments.

## Introduction

The process of fossilisation offers only a partial insight into past environments: the morphological features of organisms are preserved, but their biotic interactions and their surrounding palaeoenvironments are rarely directly observable, except in a few specific cases. Consequently, comparison with extant species is the only tool that palaeontologists usually have to understand past environments. However, the ecology of extant species may differ from that of their fossil relatives. An example is the polychelidan lobsters; a group of decapod crustaceans characterized by having four to five pairs of claws. From their first occurrence in the Triassic to Jurassic, they had well-developed eyes and occurred in various environments and depths^1^, while the few extant species all have reduced eyes and live in deep waters worldwide^2,3^.

Beurlen^4^ and Ahyong^3^ proposed that polychelidans through their evolution have shifted bathymetric ranges from shallow to deep waters. Nevertheless, the evolutionary history of polychelidans, their palaeobathymetry, and their visual systems are complex. Indeed, two distinct visual systems, apposition and reflective superposition, occur within polychelidans^5^, and while many species are reported from shallow waters, some of the earliest species inhabited deep waters^6,7^. To understand the evolutionary history of the group through time it is of utmost importance to examine the environment of each species and their phylogenetic relationships.

The Middle Jurassic La Voulte-sur-Rhône Lagerstätte (Callovian: *ca* 165 Ma) is one of the most diverse and prolific localities for polychelidan lobsters in the world. Its palaeoenvironment has been interpreted as deep-water to bathyal (more than 200 meters), possibly at the transition between the continental slope and the basin^8,9^ (see also geological context in the Supplementary Information). However, recent observation of the eyes of a thylacocephalan from La Voulte suggested that the palaeoenvironment was possibly well-illuminated and therefore shallower^10^. Here we expand on the palaeoecology and bathymetry of the La Voulte biota based on the exceptional preservation of eyes bearing ommatidia in the polychelidan lobster *Voulteryon parvulus* Audo *et al*. 2014, and a unique colonization by minute brachiopods: an unusual case of association between brachiopods and a motile host^11,12^. This association has no direct modern counterpart, and it might be linked to the higher brachiopod diversity in the Callovian than nowadays.

## Results

### Morphology of eyes

**Figure 1:**
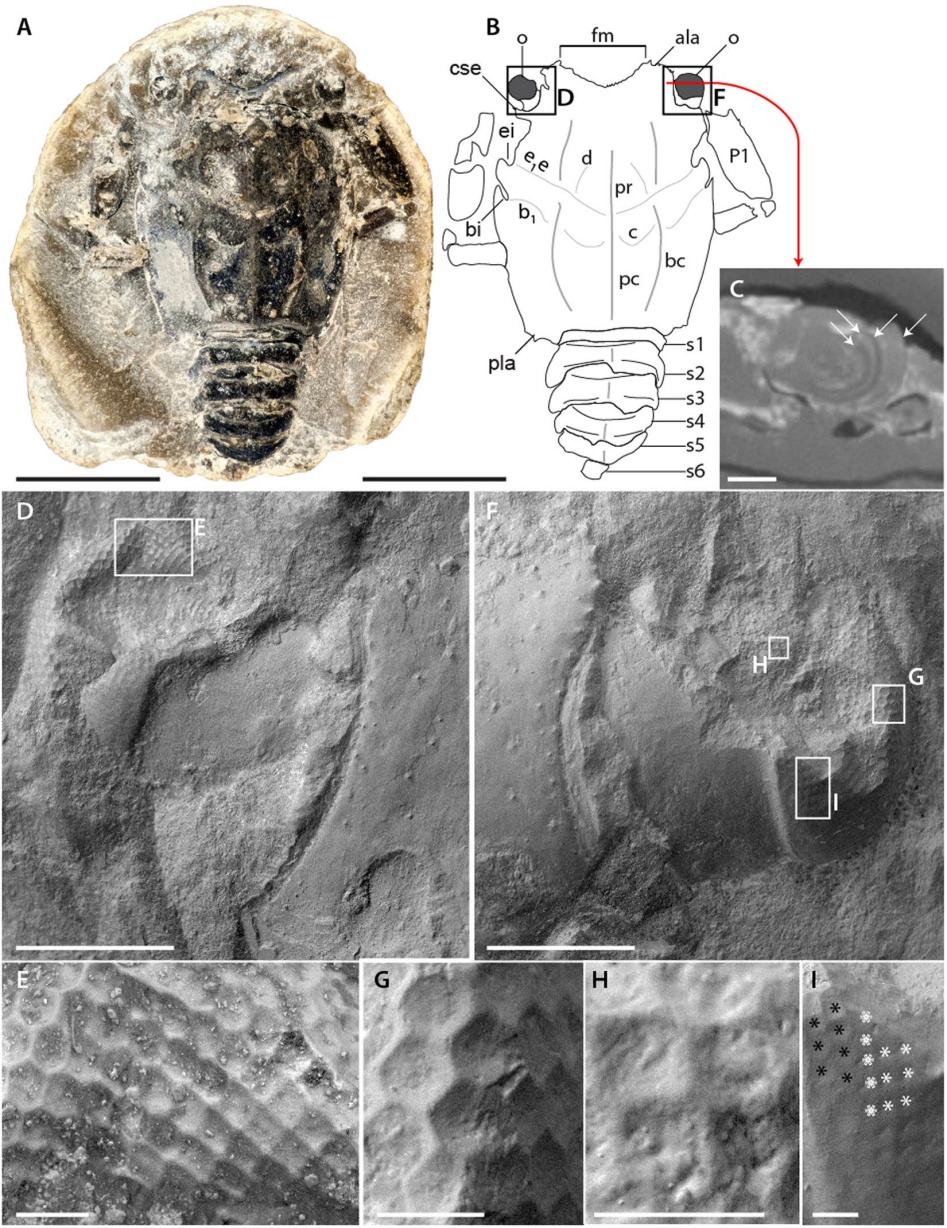
Eyes of the holotype of *Voulteryon parvulus* Audo, Schweigert, Saint Martin & Charbonnier, 2014 (MNHN.F. A50708): (**A**,**B**) complete specimen, in dorsal view, under cross-polarized light (**A**) and interpretative line-drawing (**B**,**C**) virtual slice at the level of the eye (X-ray tomography data), arrows highlight the boundaries between the various internal structure of the eye; (**D**) dorsal surface of left eye showing ommatidial lenses arrays (SEM); (**E**) transition between hexagonal and quadrate arrays with imprints of ommatidial lenses (SEM); (**F**) dorsal surface of right eye showing imprints of ommatidial lenses arrays (SEM); (**G**) hexagonal array with imprints of ommatidial lenses (SEM); (**H**) quadrate array with imprints of ommatial lenses (SEM); (**I**) more abrupt transition between hexagonal (black asterisks) and quadrate (white asterisk) arrays with imprints of ommatidial lenses (SEM). Abbreviations: ala, anterolateral angle; b_1_, hepatic groove; bc, branchial carina; bi, hepatic incision; c, cervical groove; cse, short expansion of carapace; d, gastro-orbital groove; e_1_e, cervical groove; ei, cervical incision; fm, frontal margin; o, eye; P1, pereiopod 1 (=thoracopod 4); pc, postcervical carina; pla, posterolateral angle; pr, postrostral carina; s1-s6, pleonite 1–6. Scale bars: 5 mm (**A,B**) 0.5 mm (**C**,**D**,**F**) 50 µm (**E**,**G**–**I**). Images: Denis Audo (**A**,**B**, **D**–**I**) Miguel Garcia Sanz (**C**).

**Figure 2:**
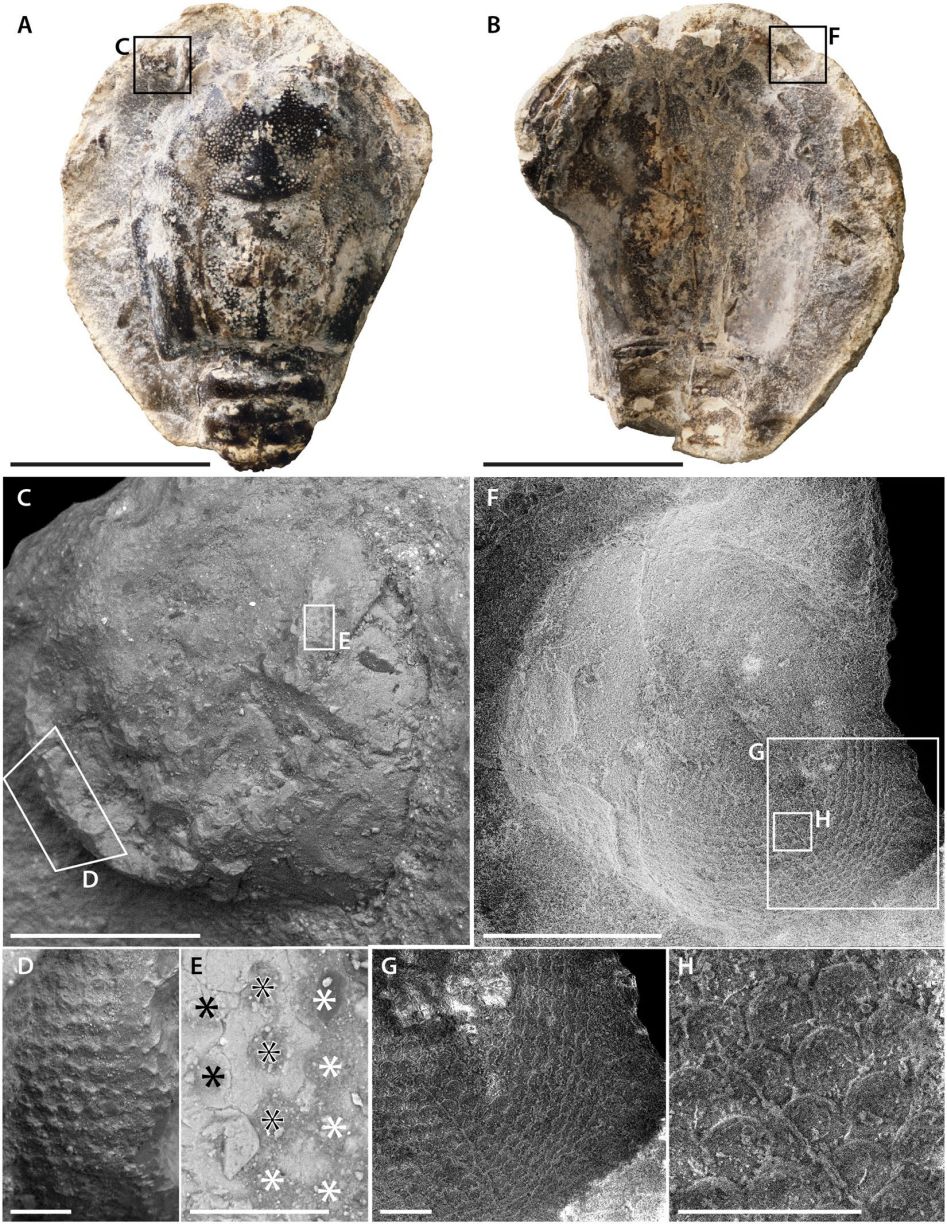
Eyes of the paratype of *Voulteryon parvulus* (MNHN.F.A29151): (**A**,**B**) complete specimen, in dorsal view, under cross-polarized light with part (**A**) and counterpart (**B**,**C**) dorsal and lateral surface of left-eye showing some preserved arrays with imprints of ommatidial lenses (SEM); (**D**) hexagonal array with imprints of ommatidial lenses (SEM); (**E**) eroded area of the eye showing what may be section of distal rhabdoms^14^, possibly at the transition between hexagonal (white asterisks) and quadrate arrays (black asterisks), the middle row appears to be intermediate (black and white asterisks) (SEM); (**F**) dorsal and lateral surface of right-eye showing some preserved arrays of ommatidial lenses (SEM); (**G,H)** hexagonal array of ommatidial lenses (SEM). Scale bars: 5 mm (**A**,**B**), 0.5 mm (**C**,**F**), 0.1 mm (**D**,**G**,**H**), 50 µm (**E**). Images: Denis Audo (**A**–**E**) and Ninon Robin (**F**–**H**).

The ocular incisions of *Voulteryon parvulus* are excavated on short expansions of the carapace reminiscent of the disposition of the eyes in *Eryon* Desmarest, 1817, and surround most of the ocular peduncle. The eyes have a long stalk ending in a short cylindrical section and a hemispheric cornea covered by hexagonally-packed ommatidia. Observation of facets is possible on the part of the holotype (Fig. 1) and on the counterpart of the paratype (Fig. 2).

#### Holotype

**Figure 3:**
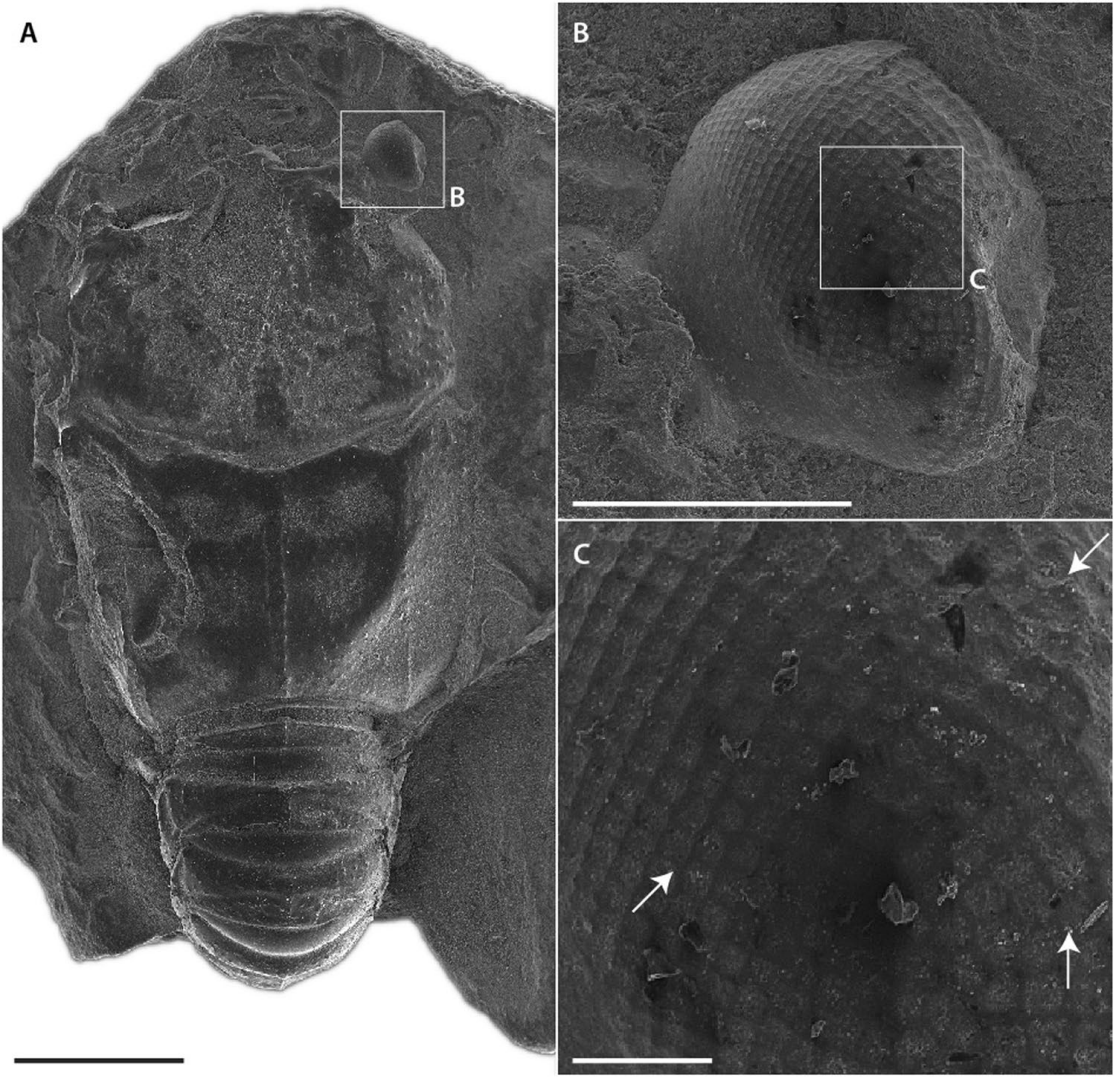
Small specimen of *Hellerocaris falloti* (Van Straelen, 1923) (MNHN.F.A50709): (**A**) entire specimen (SEM); (**B**) right eye showing imprints of ommatidial lenses (SEM); (**C**) imprints of ommatidial mostly in quadrate arrays, but also with some irregularities (indicated by arrows) (SEM). Scale bars: 2 mm (**A**), 0.5 mm (**B**) and 0.1 mm (**C**). Images: Philippe Loubry.

The left eye is incompletely preserved (Fig. 1A). The right eye diameter is about 0.95 mm (Fig. 1B). Some internal structures of ommatidia (crystalline cones, retina?) are probably preserved, but not recognized at the present time. On the tomographic virtual slices, we can only observe indistinct layers, so it is impossible to recognize if histological details similar to those observed on thylacocephalans^10^ are preserved without breaking the specimen (Fig. 1C). All the facets are made visible by the hollow traces left by the corneal lenses. This type of preservation seems frequent and can be seen in other fossil polychelidans (Fig. 3), isopods, and insects^5^. The eyes appear to have small hexagonal facets (~34 µm diameter) on most of their surfaces (Fig. 1D–G,I), although some square facets in nearly orthogonal array (with facets side ~24 µm long) are also visible dorsally (Fig. 1E,H,I). The dorsal patch of square facets is separated from the dorsal margin of the visual surface – adjacent to the stalk – by two rows of hexagonal facets (Fig. 1I), resulting in a sharp transition between hexagonal and square facets. In contrast, on the lateral region of the visual surface, the transition between square and hexagonal facets appears more gradual (Fig. 1E).

#### Paratype

The left eye diameter is about 1.15 mm (poorly preserved anteriorly) (Fig. 2A,B). As in the holotype, most of the ommatidia are hexagonally-packed and bear small hexagonal facets (~35 µm diameter) (Fig. 2C,E–H). On the part (Fig. 2A,C–E), the few ommatidia visible on the lateral region have hexagonal facets (as indicated by the imprint of the corneal lenses), although the anterior region of the eye reveals faint traces of what might be the transition between ommatidia packed in hexagonal array to a more rectilinear fashion (Fig. 2E). On the counterpart, corneal lenses are well preserved and with circular facets arranged hexagonally (Fig. 2F.H). As described for the larvae of *Palaemon serratus* (Pennant, 1777), it appears that packing is hexagonal, but that the corneal lenses themselves are circular^13^. On an eroded part of the eye (Fig. 2E), we can observe traces of ommatidia far apart from each other. This might correspond to a deeper part of the ommatidia, possibly the distal rhabdom^14^.

### Epibionts

**Figure 4:**
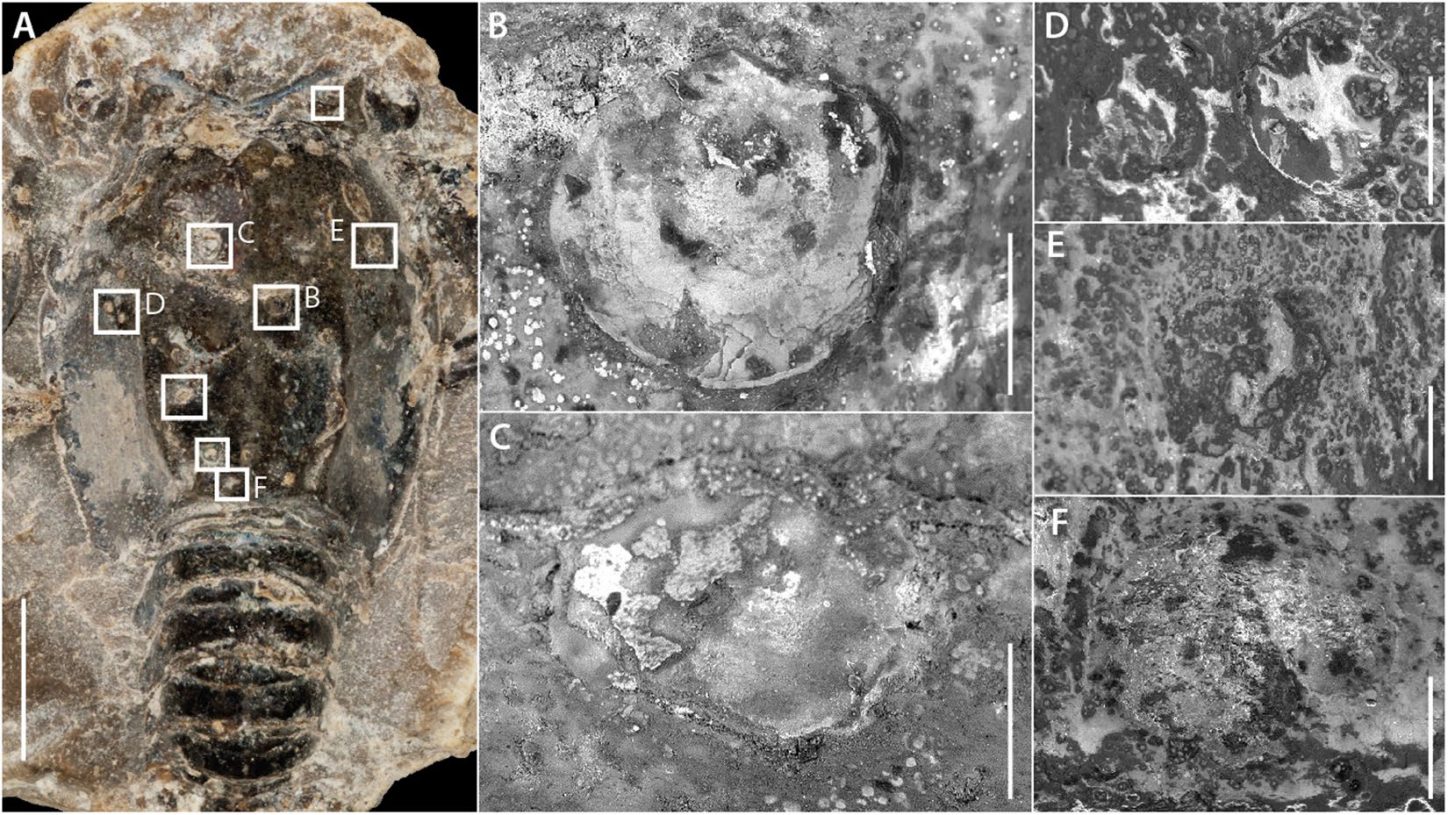
Thecidean brachiopods attached to the holotype (MNHN.F. A50708) of *Voulteryon parvulus*: (**A)** locations of eight brachiopod shelly remains; (**B**) ventral valve of the most complete brachiopod; (**C**), a second valve; (**D**) two cemented valves remains or attachment scars; (**E**,**F**) shell-shaped swellings that could correspond to damaged previously attached valves. Scale bars = 5 mm (**A**); 0.2 mm (**B**–**F**). Images: Denis Audo (**A**,**B**) and Ninon Robin (**C**–**F**).

The holotype displays on its carapace eight complete valves, shelly remains, or attachment scars of brachiopods (Fig. 4A). The most complete brachiopod, exhibiting a well-preserved ventral valve, is attached on the right side of the lobster near the median line, immediately posterior to the cervical groove (Fig. 4B). Three other epibiotic structures may correspond to the discoid attachment scars of dorsal valves. They occur near the ocular incision (Fig. 4A) anterior (Fig. 4C) and posterior (Fig. 4D) to the postcervical groove, on the left side of the specimen. Two other scars, located posterior to the branchiocardiac groove on the anterior right side of the carapace (Fig. 4E) and near the posterior margin (Fig. 4F), evoke shell-shaped surface swellings that would correspond to highly abraded ancient valves. These remains range from 180 to 500 µm in diameter.

The brachiopods attached to the specimen of *Voulteryon parvulus* have a characteristic sub-circular to sub-triangular outline (Fig. 4B,C), a feature typical of thecideid brachiopods^15,16^. Unfortunately, the diagnostic characters used in the systematics of these brachiopods are generally located on their dorsal (=brachial) valves and not ventral (=pedicle) ones, which are preserved in the specimens here discussed. In addition, the diameters of the ventral valves (or their traces – Fig. 4D–F) suggest that these brachiopods were juveniles. Due to their preservation and development stage, precise determination of specimens is impossible. Indeed, according to Baker^17^, early juveniles are not only less common than adults in the fossil record, but they are also more difficult to identify^16–18^. Nevertheless, the right antero-lateral portion of the best available specimen (Fig. 4B) preserves the exposed edge, which is typical of the ventral valves of early juvenile thecideoid shell^16^. Based on such criteria we consider these specimens as *Rioultina*-like forms. These thecideoid brachiopods were abundant during the Jurassic, with a range spanning from late Bathonian to late Oxfordian^18,19^.

## Discussion

### Size of eyes and ommatidia

**Figure 5:**
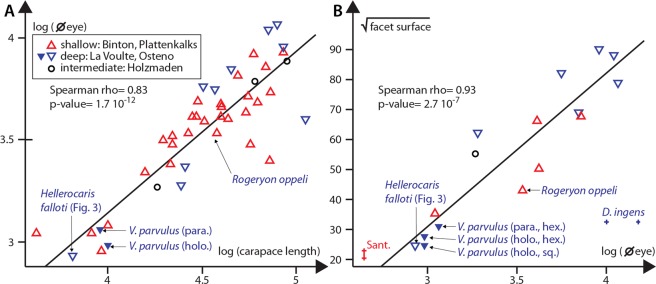
Statistical analysis of the size of the eye and ommatidia of *Voulteryon parvulus* compared to those of other fossil polychelidans and arthropods: (**A**) relation between the size of carapace and diameter of the eye in polychelidan lobsters. (**B**) relation between the size of the eye and of ommatidia in polychelidan lobsters, with a comparison to the eyes of supposed phyllosoma larvae from Santana (Sant.) and a thylacocephalan (*Dollocaris ingens*) from La Voulte. The original measurements are in micrometers. Abbreviations: holo, holotype; para, paratype; hex, hexagonal ommatidia; Sant., phyllosoma from the Cretaceous Santana Formation sq, square ommatidia;. Original data for *V. parvulus* from the present study; for polychelidans, from Audo *et al*.^5^; for phyllosoma, from Tanaka *et al*.^34^; for *Dollocaris ingens*, from Vannier *et al*.^10^.

Representatives of *Voulteryon parvulus* and other small polychelidans have small eyes and facets when compared to larger polychelidans (Fig. 5). These characteristics are unlikely to be directly linked to the environment, as Audo *et al*.^5^ showed a dominant positive correlation of eye and facet size with the carapace length. A small phylogenetic effect is possible, since eryonids tend to have smaller eyes than coleiids, the other main polychelidan clade^5^.

### Interpretation of ommatidia packing and facet shape

#### Hexagonal facets

The hexagonal facets packed in hexagonal lattice of *V. parvulus* are typical of apposition eyes, refractive and parabolic superposition eyes.

If we consider the apposition hypothesis: Eyes with apposition optics correspond to the simplest type of compound eyes, are a plesiomorphic condition for eucrustaceans, and are present in fossil and extant decapod larval stages^13,20,21^. Adult decapods in most shrimp, lobster, galatheoid anomuran, and ancient brachyuran groups, however, share a unique reflecting superposition visual system coupled to square-shaped ommatidial cornea, not seen in other marine eucrustaceans^22–25^. Noticeably, a number of decapod lineages have independently retained the larval apposition eyes^20,26^, or have evolved refractive or parabolic superposition eyes while retaining a hexagonal packing^27^. Previously, *Rogeryon oppeli* (Woodward, 1866) was the only polychelidan considered to have hexagonal facets (most likely apposition eyes) retained due to paedomorphic, more precisely neotenic evolution^5^. *Voulteryon parvulus* is probably also a paedomorphic form^28^, so it is more parsimonious to consider that its hexagonally-packed ommatidia correspond to larval apposition optics retained in adulthood as in *R. oppeli*. In contrast to *R. oppeli*, however, the adults are smaller in *V. parvulus*, and either progenesis or a combination of progenesis and neoteny may have occurred in this case. Another process might explain the presence of apposition eyes; in small scarabeoidea beetle, the eye is sometimes so small (less than 0.6 mm in diameter) that superposition optics cannot be accommodated^29^. For *V. parvulus*, such an interpretation is unlikely because: (1) a small specimen of *Palaeopentacheles roettenbacheri* (Fig. 3) has smaller eyes which are probably of the reflective superposition type^5^ yet are smaller than those of *V. parvulus* and (2) *V. parvulus* has eyes that are more than 50% bigger than the biggest eyes of the aforementioned scarabeoidea.

It is also possible, yet probably less parsimonious, that *V. parvulus*, and perhaps also *Rogeryon oppeli*^5^, had a type of superposition optics that did not require square facets: refractive or parabolic superposition optics. From the fossil alone we cannot distinguish between these two options. However, while we can propose a simple evolutionary scenario explaining the presence of apposition optics in an adult by heterochronous retention of the larval apposition eyes, having refracting or parabolic superposition optics, although likely, is less parsimonious than the retention of apposition optics.

#### Square facets

The eyes of the holotype (and likely the paratype) show small patches of square-facetted ommatidia (Fig. 1E,H). These facets with rectilinear packing are strongly reminiscent to those seen in decapods with reflective superposition eyes^26^ (although this shape can sometimes occur in parabolic and refracting superposition eyes^27,30^). These ‘mirror’ eyes are well suited for dim-light conditions, and are commonly seen across nocturnal or deep-water decapod taxa^31^.

#### Association of square and hexagonal facets

The combination of two types of structured arrays is unusual: in polychelidans, most documented arrays are square (9 species), with only one other example of hexagonal arrays^5^. Even within decapods, eyes combining hexagonal and square facets are extremely rare. They have only been reported in a handful of taxa: (1) the post-zoeal stage with all pleonal appendages of the hydrothermal vent caridean shrimp *Rimicaris exoculata* Williams & Rona, 1986^32^; (2) the adult benthesicymid shrimp^30^; (3) isolated decapod fossil eyes, supposedly of a phyllosoma larva from the Santana Group^33^; and (4) at least one extinct and three extant brachyuran species^34^ (Luque, personal observation). Yet, it is unclear whether such a combination of facet shape and packing implies that two discrete and functional visual systems are present (which may need different underlying optical neuropiles), or if it may occur as the outcome of the packing, size, and position of marginal facets being accreted to the eye.

Another fossil with an apparent combination of facet types is the polychelidan lobster *Hellerocaris falloti* (Van Straelen, 1923), also from La Voulte (Fig. 3). In *H. falloti*, most of the facets are square and packed orthogonally as in reflecting superposition eyes, although some facets are hexagonal or irregular (Fig. 3C). However, the non-square facets in *H. falloti* may just be a local packing artifact rather than a true regionalization of the eye. Contrary to *H. falloti*, in *V. parvulus* hexagonal facets are the dominant type, and may correspond to larval apposition optics retained in later instars, with the discrete squarish facets likely representing either a) an artefact of the packing, or b) a transition towards the adult reflecting superposition eye type seen across most of polychelidans from La Voulte.

In general, the number of ommatidia increases with growth, and the ommatidia may be inserted from a dorsal accretion zone, which points to a gradual development of the adult eye from the larval eye^13,35^. In the case of mantis shrimps, the adult optics of the eye arise on the ocular peduncle separated from the larval optics of the eye, and pushe it aside as they develop^35,36^. In the case of the gradual development of the eye, it seems that ommatidia optics are “pre-adapted” to their adult forms: light is reflected or refracted as in superposition optics, but the lack of clear zone and adaptations of the rhabdom force the eye to function as an apposition eye. This scenario is similar to that proposed by Tanaka *et al*.^33^ to explain the occurrence of a patch of square facets on the anterior part of isolated fossil eyes.

Another interesting possibility is presented by benthesicymid shrimps, where the visual surface can be composed of more than one third of square facets, the rest being hexagonal. It has been proposed that these square facets are an evolutionary relict of ancestral reflective superposition optics^30^. Such a case would not be too surprising in the case of *V. parvulus* since the presence of reflective superposition optics is the ancestral condition in polychelidan lobsters is the presence of reflective superposition optics.

### Visual capacities of the eyes in a deep-water environment

*Voulteryon parvulus* differs from most other polychelidans and glypheidans from La Voulte, which are very likely to possess reflective superposition eyes^5^ (see also Charbonnier *et al*.^37^, Fig. 337). Besides, due to its small size, the corneal lenses cannot compensate the absence of superposition optics by their size (larger ommatidial lenses can collect more light, as in deep-sea isopods or anomura, which can have large ommatidial lenses^25,38^ – but also consider that small ommatidial lenses in *V. parvulus* are expected, since size of ommatidial lenses is mostly proportional to the size of the animal^5^). The pooling of ommatidia signal as it occurs in deep-water hyperiid amphipods^39^ also seems unlikely, since hyperiids have bilobed eyes (i.e., one lobe with a higher resolution and another with higher sensitivity) absent in *V. parvulus* or other polychelidan lobsters. Yet, we cannot exclude other processes that would increase dramatically the sensitivity of the eye such as temporal summation (photons are collected over longer periods of time, at the expense of temporal resolution) or neural resolution (adjacent rhabdoms combine part of their signal by neural connection^40^, herein impossible to study).

From the available data, we infer that the eyes of *V. parvulus* were likely poorly adapted to dim-light environments, as expected from eyes of the apposition type (note however that some possible mechanisms to increase sensitivity such as tapetum or rhabdom adaptations cannot be observed). Yet, whether the small square-facetted area in the eyes of *V. parvulus* functioned as superposition optics or not is unlikely, since superposition optics necessitate numerous ommatidia to collect the light optimally. Contrary to most other polychelidans and glypheidans in La Voulte, and similarly to *Rogeryon oppeli*, *V. parvulus* might have been better suited for bright-light conditions. This is surprising in a disphotic palaeoenvironment as it has been proposed by Charbonnier *et al*.^9,41^ for La Voulte. In fact, the size of corneal facets and apposition optics of *V. parvulus* are similar to that of the co-occurring thylacocephalan crustacean *Dollocaris ingens* Van Straelen, 1923, which was considered to be adapted to well-illuminated environments.

We also acknowledge that it is possible that *V. parvulus* had a type of superposition optics that does not require square facets. i.e., refractive superposition, parabolic superposition. As above explained, this hypothesis is slightly less parsimonious, but it would however explain the presence of *V. parvulus* in a supposedly deep-water palaeoenvironment.

### Ontogeny

Despite its small size (holotype carapace maximum length = 10.0 mm, paratype carapace maximum length = 9.1 mm), *Voulteryon parvulus* was at a sexually mature size as attested by the development of the ovaries in the holotype^28^, and thus considered as paedomorphic. The development of the visual surface in *V. parvulus* herein documented reinforces the interpretation that *V. parvulus* was paedomorphic. If we consider that its ommatidial array is ontogenetically intermediate between larval and adult eye, it is surprising to observe such a late replacement of apposition optics. Even small juvenile specimens of *Palaeopentacheles roettenbacheri* (Münster, 1838) and *Hellerocaris falloti* have fully developed reflective superposition eyes^5^. The apposition optics of the eye of *V. parvulus* might therefore represent a case of neoteny, just as in *Rogeryon oppeli*, with the added minute size of specimens. It is possible that despite being sexually mature, *V. parvulus* had not finished its growth. Indeed, other eucrustaceans continue to grow after their sexual maturity^42^.

### Epibionts

#### Age of the epibionts

Contrary to the well documented size-frequency patterns in fossil and modern brachiopods^43–45^, data reporting precise size/age ratio are far less available, especially for very young shell stages. Indeed, very small brachiopods are rarely recovered as fossils^46^ and modern early instars are difficult to survey because of their small size. They often display transparent valves, and have a propensity for cryptic settlement^47^.

The age of the epibiotic brachiopods on *V. parvulus* (Fig. 4) can be assessed from measurements obtained from communities of modern benthic and subtidal temperate species (Scotland and New Zealand, respectively), and on one Antarctic species whose growth occurs much slower than that of temperate ones. Extrapolating data obtained on post-larval terebratulids^48^, we infer that the largest fossil brachiopods associated to *V. parvulus* attached and grew to their size in a timeframe of more than a month (500 µm reached in 43 days).

The exquisite preservation of the polychelidan host with appendages and delicate structures such as the eyes, all preserved in connection but above the internal organs fossilized in 3D^28^, implies a very short *post-mortem* exposure of its carcass. The inferred rapid burial of the host body (or displacement of the carcass to an environment unfavourable to many organisms) contradicts any *post-mortem* attachment/growth of the brachiopods that would have lasted more than a month. Thus, the brachiopods most likely colonized *V. parvulus* while alive, further supported by the location of the thecideoids on the crustacean, all attached to the carapace and none on the more articulated segments of the pleon (Fig. 4). This selective distribution evokes a biological elimination of the brachiopods. Indeed, attachment of the young epibionts to the pleonites should have been limited by the constant movements along the pleonal articulations.

#### Rarity of the association

Due to their sessile habit, brachiopods may be expected to live onto many other hard-bodied organisms. However, cases of fossil brachiopods identified as ancient ectosymbionts are few. Most examples known so far correspond to Cambrian associations from the Burgess shale-type deposits and Mesozoic sponge-reef foulings. Indeed, symbiotic lingulate brachiopods are known to have colonized the frond of the Cambrian algae-like organisms *Malongitubus*^49^ and the spicules of sponges (Paterinata and Kutorginata)^50^. Nisusiid brachiopods are known to have been living in commensal of the motile *Wiwaxia corrugata* Walcott, 1911, attached to the dorsal spine of these Cambrian possible molluscs^12^. They are also reported as ectosymbionts of other lophophorates, like other brachiopods from the Cambrian of China^51^ and Canada^52^ or the Silurian of England^53^; but also possibly attached to the disk of the middle Cambrian eldonioid *Rotadiscus guizhouensis* Zhao & Zhu, 1994, whose systematic nature and life habits remain poorly understood (semi-motile?)^54^. Spiriferid brachiopods attached on the proximal part of echinoderms spicules from the Carboniferous of Texas were also identified as likely commensals of their hosts^55^. From the Jurassic, some studies attempted to demonstrate the *syn-vivo* nature of some large scale foulings-like on sclerosponges^19,56^, bivalves^57^ or other brachiopods (lacunosellids)^19^.

Evidences of clear *post-mortem* associations have also been identified; they involve a “stem-group” of brachiopod on the carcasses of the Cambrian arthropod *Sidneyia inexpectans* Walcott, 1911^58^ but also much more recent thecideoids on the inner mould of Paleocene crabs^59^.

Consequently, confident reports of fossil symbiotic brachiopods on motile organisms were hitherto restricted to the colonization of Wiwaxiidae (possible molluscs), and to typical Burgess-like environments and fauna. The association of Jurassic thecideoid brachiopods and a polychelidan lobster here described corresponds to novel association between brachiopods and a motile benthic host (see Table 1 in the Supplementary Information). The colonisation of such a substrate may have provided various benefits to the thecideoids: (1) obtaining an attachment on a hard substratum; (2) avoiding being silted by sedimentary supplies; (3) access to an increased food supply for such filter-feeders provided by the locomotion of their host; (4) obtaining a favoured dispersion of the larvae, and thus a wider range of potential substrates to colonise^60^.

In the Ravin des Mines, where the Lagerstätte was deposited and *V. parvulus* was discovered, the substrate was soft (see Geological context in the Supplementary Information). Therefore, at this place, thecideoids were more likely to attach to other organisms. On the other hand, the Ravin du Chénier, a fossil locality without exceptional preservation, contains a rather rich sessile bathyal fauna involving a diversity of hexactinellid sponges and crinoids, ranging from the disphotic to aphotic portion of the continental slope, suggesting the presence of a hard substrate^8,41^. Surprisingly, in the latter, epibionts including thecideoids are rare^41^, and this fouling competition must have been inferior to that occurring in circalittoral environments. Given that the brachiopods presence should not have hindered the host fitness in any significant way, a commensal and facultative interaction between these thecideoids and *V. parvulus* can be inferred. This relation displays no counterpart reported in modern environment. That uniqueness could result of the under-examination of modern marine arthropods for their epibionts, like observed for other taxa (e.g. foraminiferans and bivalves^60,61^, but also of the reduced diversity of extant brachiopods.

#### Palaeobiology of the epibiont

Thecideoid brachiopods are small cementing articulated cavity-dwelling forms. In modern environments, they occupy dim-light environments of cryptic habitats (=cryptobionts or coelobites^62^ – *sensu* Kobluk^63^), and the most probably colonised similar habitats in the past^44,64–67^). More precisely, in modern environments, they are typical for shallow water crevices, submarine caves, undersides of sponges and/or corals or other marine invertebrates and are widely distributed in tropical and subtropical seas^64,68–70^ at a depth ranging from a few meters to about 150 m^68,71–76^). In the fossil record, thecideoid brachiopods often used lower surfaces of sponges as hard substrate^19,56,77^, coral-reef cavities^78^, empty shells on the sea floor^79^, and small cryptic spaces resulting from the disintegration of hardgrounds^62,80–84^. They also incrusted surfaces of Jurassic brachiopods (Oxfordian lacunosellids^19^), bivalves (Kimmeridgian oysters *Actinostreon*^85^; Callovian *Ctenostreon*^57^), sclerosponges (Oxfordian *Neuropora*^19^), oncoids (Bajocian^86^ and Bathonian^87^) or bored cobbles^83,88^. Exceptionally, some extant thecideoid brachiopods live in deep-water environments at depths between 400 and 1000 m^76^.

The cavity-dwelling strategies of cryptic brachiopods have been used earlier, not only in Mesozoic times (e.g. Triassic *Thecospira*^89–91^), but probably also in the Palaeozoic (e.g., Silurian *Leptaenoidea* and *Liljevallia*^92^, Devonian *Davidsonia*^93^), which have close phylogenetic relationships with thecideoid brachiopods^94^. Therefore, these thecideoid epibionts on the carapace of *V. parvulus* are a rare example of using mobile benthic animals as hard substratum for their colonisation. This contrasts with the standard occurrences of these brachiopods on sessile benthic invertebrates or pieces of rocks. Additionally, their photophobic character indicates at least dark palaeoenvironments during incrustations and can be used to infer bathymetric conditions of their host, deeper than photic zone of the sea floor.

## Conclusion

The exceptional preservation of *Voulteryon parvulus* from the Middle Jurassic of La Voulte, France, including the presence of compound eyes bearing facets (this work), a unique example of a *syn-vivo* association between epibiont brachiopods and crustaceans (this work), and ovaries^28^, gives new insights on the palaeoecology of this polychelidan lobster and the La Voulte Lagerstätte.

The epibiont brachiopods of *V. parvulus* generally inhabit dim-light environments, which is congruent with previous hypothesis by Charbonnier *et al*.^41^ and Charbonnier^8^ of deep-water, low-lit conditions during the deposition of the La Voulte Lagerstätte. Surprisingly, the most parsimonious interpretation of the surface of the eyes in *V. parvulus* suggests limited capacity for vision in low-light conditions. These two apparently opposed results are difficult to explain with certainty. One hypothesis is that La Voulte Lagerstätte was not as deep as what seems to be indicated by the geology and sponges in the nearby Chénier ravine (hypotheses of Charbonnier *et al*.^41^ and Charbonnier^8^). This would agree with Vannier *et al*.^10^, who suggested more lit conditions based on the presence of the large-eye predatory arthropod *Dollocaris ingens*. Yet, the presence of thecideoid brachiopods on *V. parvulus* strongly indicates dim-light conditions: either due to the depth in La Voulte or to the microenvironment of crevices in a shallower environment.

**Figure 6:**
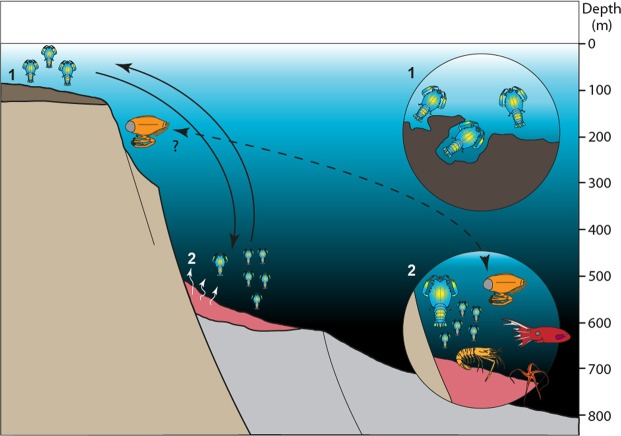
Visual depiction of the palaeoenvironment of *Voulteryon parvulus*: *Voulteryon parvulus* lived probably in a shallow-water, well-illuminated environment, perhaps in crevices (1), explaining the presence of its epibionts and perhaps used to mate or lay its eggs in the La Voulte Lagerstätte (2). Note that due to the presence of numerous faults, the palaeotopography of the vicinity of the La Voulte Lagerstätte was quite accidented. Therefore, *Voulteryon parvulus* did not have to move over great distances to reach deeper waters. Similarly, *Dollocaris ingens* could as well move or be transported in La Voulte deposition environment from a nearby shallower palaeoenvironment.

*Voulteryon parvulus* was a mobile organism, and the geology in the vicinity of La Voulte is dominated by numerous faults that probably created an irregular bathymetry at the time of deposition of the La Voulte Lagerstätte. We therefore propose two additional hypotheses that could explain all observations (Fig. 6):

1. *Nature of the eyes:* Adults of *V. parvulus* had refractive or parabolic superposition eyes, so they were capable of living in a dim-light environment. The only problem of this hypothesis is that it requires an independent evolution of parabolic or refractive superposition eyes from either reflective superposition or apposition eyes.
2. *Displacement or natural migration: V. parvulus* may have lived in a more well-illuminated environment (as suggested by its eyes); which probably would have been different from the one in La Voulte. Since thecideoid brachiopods prefer crevices or dim light, it is possible that *V. parvulus* used to hide in rock crevices; *V. parvulus* was then either flushed to the deposition environment of La Voulte, or, more probably, La Voulte could have been a reproductive ground for *V. parvulus*^28^. This last suggestion seems plausible but opens the question: did *V. parvulus* spend enough time in the depth of La Voulte for the fixation and growth of the epibionts? Unfortunately, too little is known about the reproductive behaviour of extant polychelidans^95,96^ to compare it with their fossil relatives.

Transportation of the thylacocephalan *Dollocaris ingens*, facilitated by its nektobenthic lifestyle^10^, and the irregular palaeobathymetry in the La Voulte area, could also explain the apparent mismatch between geological data and the ecology of some arthropods as suggested by their exceptionally preserved visual systems. La Voulte biota clearly combines the influence of multiple palaeoenvironments, as expected from the complex local geology.

## Material and Methods

### Material

We study the only two known specimens of *Voulteryon parvulus* (holotype: MNHN-F.A50708; paratype: MNHN.F.A29151), from the La Voulte-sur-Rhône Lagerstätte, housed in the palaeontology collection of the Muséum national d’Histoire naturelle (MNHN.F specimens). Both specimens are three-dimensionally preserved inside sideritic nodules and show very fine anatomical structures^1,28^. The holotype consists only of the part, and the paratype consists of both part and counterpart.

### Imagery techniques

Global views of the holotype and paratype of *Voulteryon parvulus* were done using a cross-polarized light setup^97,98^) coupled to image stacking. Colour detailed views of the epibionts and eyes on the holotype were obtained with a 24 × 36 mm digital SLR equipped with a x10 microscope objective. The holotype was micrographed using a Hitachi Analytical Table Top Scanning Electron Microscope. The paratype was micrographed using a Tescan SEM (VEGA II LSU) linked to an X-ray detector SD3 (Bruker) (Direction des Collections, MNHN, Paris).

Additionally, we used the virtual slices and the reconstruction of the ventral surface of the holotype by Jauvion *et al*.^28^, reconstructed using the VG-Studio MAX 2.2 (© Volume Graphics) software on X-Ray Tomography (XTM, CT-Scan) data (voxel size = 9.55 µm, 2007 virtual slices), acquired at the AST-RX platform (USM 2700, MNHN) on a v/tome/x 240 L tomograph (GE Sensing & Inspection Technologies Phoenix X/ray) and equipped with a microfocus 240 kV/320 W tube delivering a current/voltage of 485 mA/95 kV.

## Supplementary information

Supplementary Information

## Data Availability

All data needed to evaluate the conclusions in the paper are present in the paper and/or the Supplementary Information.
