# Supplementary material for: Palaeoecology of Voulteryon parvulus (Eucrustacea, Polychelida) from the Middle Jurassic of La Voulte-sur-Rhône Fossil-Lagerstätte (France)
Source: Sci Rep. 2019 Mar 29;9:5332. doi: 10.1038/s41598-019-41834-6 (PMC6441058; doi:10.1038/s41598-019-41834-6)
Supplement: Supplementary file 1 — Supplementary Information [file 41598_2019_41834_MOESM1_ESM.docx]

Supplementary information

# Geological context

The La Voulte Lagerstätte (Middle Jurassic, Callovian) is located in south-eastern France (Ardèche) along the right bank of the Rhône River (150 km south of Lyon) and belongs to the eastern sediment cover of the Massif Central. The Massif Central is a Hercynian crystalline complex whose eastern margin has been faulted and tilted during the Middle Jurassic. A major sub-vertical normal fault, the so-called La Voulte fault (N54°, inherited Hercynian direction) used to be very active during the Callovian and cuts through the whole area. The most recent palaeogeographic reconstructions for the Callovian place the La Voulte area along the western margin of the Tethys Ocean and adjacent to the Massif Central, which was probably submerged at that time^1^. The Tethyan margin running east of La Voulte was characterized by a complex submarine palaeotopography of tilted blocks generated by a series of inherited Hercynian and transverse faults. Platforms lying on the submerged Massif Central were the principal source of carbonate sediments whereas terrigenous inputs originated partially from the sub-marine erosion of the Hercynian basement and its Early Mezosoic sediment cover^2,3^. The biostratigraphy of the La Voulte area was established by Elmi^4^ and is based on ammonite biozonation: the deposits belong to the early Callovian Gracilis Biozone.

The fossiliferous layers of the La Voulte Lagerstätte crop out in the Ravin des Mines southwestern of the town of La Voulte-sur-Rhône and occur within a relatively thin interval (5–6 m). Their lithology consists of marls topped with 15 m thick iron deposits that were exploited in the 19th century^5^. The basal marls contain numerous sideritic nodules, which frequently preserve crustaceans retaining most of their original volume and more or less undeformed soft-bodied cephalopods. Some marly horizons are also locally rich in soft-bodied fossils (e.g. cephalopods, worms) that occur more flattened. Thin iron carbonate layers with abundant ophiuroids occur throughout the succession^1^. An updated faunal inventory of the La Voulte Lagerstätte was proposed in the monograph of Charbonnier^6^. With the more recent studies of Charbonnier *et al.*^7^, Audo *et al.*^8^, Jauvion *et al.*^9,10^, and Kruta *et al.*^11^, the whole fauna consists of about 70 different species among which the most typical elements are arthropods (*ca* 40 species), cephalopods (7 species, except ammonites), marine worms (7 species), echinoderms (ophiuroids and sea stars, 6 species), vertebrates (marine crocodile, and sharks, 4 species), bivalves (2 species) and brachiopods (2 species).

# Associations between branchiopods and organic substrates

Table 1. — Cases of associations between brachiopods and organic substrates that were tentatively analysed by scientists, including that of the polychelidan lobster *Voulteryon parvulus* from the Middle Jurassic of La Voulte-sur-Rhône, France.

| **Brachiopod epibiont** | **Host and location** | **Host lifestyle** | **Association** | **Age** | **Locality** | **Ref.** |
| --- | --- | --- | --- | --- | --- | --- |
| *Kuangshanotreta malungensis* (Lingulata, Acrotretoidea) | Frond of the Cambrian *Malongitubus kuangshanensis*  (Hemichordata?, Pterobranchia?, Graptolithina?) | Sessile | *Syn-vivo* | Early Cambrian | Kuangshan, Yunnan China | 12 |
| *Micromitra* (Paterinata, Paterinidae)  and  *Nisusia* (Kutorginata, Kutoriginida) | Spicules of *Pirania* (Porifera) | Sessile | *Syn-vivo* | Early  Cambrian | Burgess Shale, British Columbia, Canada | 13 |
| *Longtancunella chengjiangensis* (Chileata?) | Shell of *Diandongia pista* (Brachiopoda, Botsfordiidae) | Sessile | *Syn-vivo* | Early Cambrian | Yu’anshan, Yunnan, China | 14 |
| *Acanthotretella spinosa* (group of “stem-brachiopoda”) | Antenna of *Sidneyia* (Euarthropoda, Merostomoidea, Limulavida) | Motile | *Post-mortem* | Early Cambrian | Burgess Shale, British Columbia, Canada | 15 |
| *Nisusia* (Kutorginata,  Kutoriginida) | Dorsal spine of *Wiwaxia* (Lophotrochozoa, Mollusca?) | Motile | *Syn-vivo* | Middle Cambrian | Burgess Shale, British Columbia, Canada | 16 |
| *Nisusia* (Kutorginata,  Kutoriginida) | Skeleton of *Hazelia* (Porifera, Demonspongiae), Chancelloria (Coeloscleritophora, Chancelloriida) and tube of *Selkirkia* (Priapulida, Eupriapulida) | Sessile | Unsure | Middle Cambrian | Burgess Shale, British Columbia, Canada | 16 |
| *Nisusia? burgessensis*, *Nisusia* sp. (Kutorginata, Kutoriginida);  *Micromitra* sp. (Paterinata,  Paterinidae) and Acrotretida indet. | Helens of *Haplophrentis* (Lophophorata, Hyolithida) | Semi-sessile | *Syn-vivo* | Middle Cambrian | Burgess Shale, British Columbia, Canada; Spence Shale, Idaho & Utah, USA | 17 |
| Obollelida? indet. | Disk of *Rotadiscus guizhouensis* (Cambroernida, Eldoniidae) | Likely sessile | *Syn-vivo* and possibly *post-mortem* | Middle Cambrian | Kaili Formation; Guizhou; China | 18 |
| *Orbiculoidea* indet. (Lingulata, Lingulida, Disciniidae) | Orthocone of nautiloids (Mollusca, Cephalopoda) | Motile | *Syn-vivo* | Upper Ordovician (Ashgill) | Soom Shale, Western Cape, South Africa | 19 |
| Brachiopoda indet. (juvenile) | Shell of *Bethia serraticulma*  (Brachiopoda, Rhynchonellata? Orthida?) | Sessile | *Syn-vivo* | Silurian | Herefordshire, West Midlands, United-Kingdom | 20 |
| *Poloniproductus*? (Productida, Productidae)  and  Cyrtinitidae indet. | Stems of *Schyschcatocrinus creber* and  *Tantalocrinus scuttelus* (Echinodermata, Crinoidea) | Sessile | *Syn-vivo* | Middle Devonian (Eifelian) | Holy Cross Mountains, Poland | 21 |
| *Crurithyris* *planoconvexa* (Rhynchonellata, Spiriferida, Ambocoeliidae) | Proximal part of the spicules of *Archaeocidaris* (Echinodermata, Echinoidea, Archaeocidarididae) | Motile | Likely *syn-vivo* | Carboni-ferous | Texas, USA | 22 |
| *Rioultina triangularis* (Thecideoida, Thecideidae) | Fronds of *Platychonia* *magna* (Demospongea, Lithistida, Platychoniidae) | Unsure | Likely *syn-vivo* | Middle Jurassic (Bathonian) | Saint-Aubin-sur-mer, Calvados, France | 23 |
| Moorelina (Thecideoida, Thecideidae) | Shells of Ctenostreon proboscideum (Mollusca, Bivalvia, Limoida, Limidae) | Sessile | *Post-mortem* and possibly *syn-vivo* | Middle Jurassic (Callovian) | Zalas, Mazovia, Poland | 24 |
| Thecideidae indet. | Dorsal carapace of *Voulteryon parvulus* (Eucrustacea, Polychelida) | Motile | *Syn-vivo* | Middle Jurassic (Callovian) | La Voulte-sur-Rhône, Ardèche, France | 9, here |
| *Rioultina wapiennensis*  and  *Neothecidella ulmensis* (Thecideida, Thecideidae) | Shells of *Lacunosella cracoviense*  (Brachiopoda, Rhynchonellata, Basiliolidae), other Rhynchonellids, Terebratulids and oysters.  skeletons of Hexatinellida, “Listhida”, Calcispongea, Sclerospongea (*Neuropora* sp.), echinoids, bryozoans. | Sessile | Unsure | Upper Jurassic (Oxfordian) | Kujawy, Poland | 25 |
| Thecideoida indet. | Inner mold of the carapace of *Dromiopsis rugosa* (Eucrustacea, Brachyura) | Motile | *Post-mortem* | Early Paleocene | Fakse, Zealand, Denmark | 26 |

# References

1. Charbonnier, S., Vannier, J., Gaillard, C., Bourseau, J.-P. & Hantzpergue, P. The La Voulte Lagerstätten (Callovian): Evidence for a deep water setting from sponge and crinoid communities. *Palaeogeogr. Palaeocl.* **250**, 216-236. [10.1016/j.palaeo.2007.03.013](http://dx.doi.org/10.1016/j.palaeo.2007.03.013) (2007).
2. Elmi, S. Stages in the evolution of late Triassic and Jurassic carbonate platforms: the western margin of the Subalpine Basin (Ardèche, France). in *Carbonate Platforms, Facies, Sequences and Evolution. Special publication number 9 of the International Association of Sedimentologists* (eds Tucker M. E. *et al.*), 109-144. (Blackwell Scientific Publications ,1990).
3. Elmi, S. Les applications géodynamiques de la stratigraphie: l’histoire triasico-jurassique de la marge vivaro-cévenole (France, Sud-Est). *Documents des Laboratoires de Géologie de la Faculté des Sciences de Lyon* **hors-série 9**, 93-123 (1990).
4. Elmi, S. Le Lias supérieur et le Jurassique moyen de l’Ardèche. *Documents des Laboratoires de Géologie de la Faculté des Sciences de Lyon* **1**, 1-845 (1967)..
5. Ledoux, C. *Etude sur les terrains triasique et jurassique et les gisements de minerai de fer du département de l’Ardèche*, 1-115 (Savy, 1868).
6. Charbonnier, S. Le Lagerstätte de La Voulte : un environnement bathyal au Jurassique. *Mémoir. Mus. natl. Hist. nat.* **199**, 1-272 (2009).
7. Charbonnier, S., Garassino, A., Schweigert, G. & Simpson, M. A worldwide review of fossil and extant glypheid and litogastrid lobsters (Crustacea, Decapoda, Glypheoidea). *Mémoir. Mus. natl. Hist. nat.* **205**, 1-304 (2013).
8. Audo, D., Schweigert, G., Saint Martin, J.-P. & Charbonnier, S. High biodiversity in Polychelida crustaceans from the Jurassic La Voulte-sur-Rhône Lagerstätte. *Geodiversitas* **36**, 489-525; [10.5252/g2014n4a1](http://dx.doi.org/10.5252/g2014n4a1) (2014)
9. Jauvion, C., Audo, D., Charbonnier, S. & Vannier, J. Virtual dissection and lifestyle of a 165-million-year-old female polychelidan lobster. *Arthropod Struct. Dev.* **45**, 122-132 [10.1016/j.asd.2015.10.004](http://dx.doi.org/10.1016/j.asd.2015.10.004) (2016).
10. Jauvion, C., Charbonnier, S. & Bernard, S. A new look at the shrimps (Crustacea, Decapoda, Penaeoidea) from the Middle Jurassic La Voulte-sur-Rhône Lagerstätte. *Geodiversitas* **39**, 705-716 [10.5252/g2017n4a3](https://doi.org/10.5252/g2017n4a3) (2018).
11. Kruta, I., Rouget, I., Charbonnier, S., Bardin, J., Fernandez, V., Germain, D., Brayard, A. & Landman, N. *Proteroctopus ribeti* in coleoid evolution. *Palaeontology* **59**, 767-773 (2016).
12. Wang, H., Zhang, Z., Holmer, L. E., Hu, S., Wang, X., & Li, G. Peduncular attached secondary tiering acrotretoid brachiopods from the Chengjiang fauna: Implications for the ecological expansion of brachiopods during the Cambrian explosion. *Palaeogeogr. Palaeocl.* **323**, 60-67 [10.1016/j.palaeo.2012.01.027](https://doi.org/10.1016/j.palaeo.2012.01.027) (2012).
13. Conway-Morris, S., & Whittington, H. B. Fossils of the Burgess shale: a national treasure in Yoho National Park, British Columbia. *Geol. Surv. Canada Misc Report* **43**, 1-31 (1985).
14. Zhang, Z., Han, J., Wang, Y., Emig, C. C., & Shu, D. Epibionts on the lingulate brachiopod Diandongia from the Early Cambrian Chengjiang Lagerstätte, South China. *P. Roy. Soc. Lon B Bio* **277**, 175-181. [10.1098/rspb.2009.0618](https://dx.doi.org/10.1098/rspb.2009.0618) (2010).
15. Holmer, L. E., & Caron, J. B. A spinose stem group brachiopod with pedicle from the Middle Cambrian Burgess Shale. *Acta Zool.* **87**, 273-290 [10.1111/j.1463-6395.2006.00241.x](https://doi.org/10.1111/j.1463-6395.2006.00241.x) (2006).
16. Topper, T. P., Holmer, L. E., & Caron, J. B. Brachiopods hitching a ride: an early case of commensalism in the middle Cambrian Burgess Shale. *Sci. Rep.* **4**, 6704 [10.1038/srep06704](https://doi.org/10.1038/srep06704) (2014).
17. Moysiuk, J., Smith M.R., and Caron, J.B. Hyoliths are Paleozoic lophophorates. *Nature* **541**(7637), 394–397 [10.1038/nature20804](https://doi.org/10.1038/nature20804) (2017).
18. Dzik, J., Zhao, Y., & Zhu, M. Mode of life of the Middle Cambrian eldonioid lophophorate *Rotadiscus*. *Palaeontology* **40**, 385-396 (1997).
19. Gabbott, S. E. Orthoconic cephalopods and associated fauna from the Late Ordovician Soom Shale Lagerstätte, South Africa. *Palaeontology* **42**, 123-148 [10.1111/1475-4983.00065](https://doi.org/10.1111/1475-4983.00065) (1999).
20. Sutton, M. D., Briggs, D. E., Siveter, D. J., & Siveter, D. J. Silurian brachiopods with soft-tissue preservation. *Nature* **436**(7053), 1013-1015 [10.1038/nature03846](https://doi.org/10.1038/nature03846) (2005).
21. Gluchowski, E. (2005). Epibionts on upper Eifelian crinoid columnals from the Holy Cross Mountains, Poland. *Acta Palaeontol. Pol.* **50**,
22. Schneider, C. L. Hitchhiking on Pennsylvanian echinoids: epibionts on *Archaeocidaris*. *Palaios*, **18**, 435-444 [10.1669/0883-1351(2003)018%3C0435:HOPEEO%3E2.0.CO;2](https://doi.org/10.1669/0883-1351(2003)018%3C0435:HOPEEO%3E2.0.CO;2) (2003).
23. Palmer, T. J. & Fürsich, F. T. Ecology of sponge reefs from the Upper Bathonian of Normandy. *Palaeontology* **24**, 1-23 (1981).
24. Zatoń, M., Wilson, M. A. & Zavar, E. Diverse sclerozoan assemblages encrusting large bivalve shells from the Callovian (Middle Jurassic) of southern Poland. *Palaeogeogr. Palaeocl.* **307**, 232-244 [10.1016/j.palaeo.2011.05.022](https://doi.org/10.1016/j.palaeo.2011.05.022) (2011).
25. Krawczyński, C. The Upper Oxfordian (Jurassic) thecideide brachiopods from the Kujawy area, Poland. *Acta Geol. Pol.* **58**, 395-406 (2008).
26. Jakobsen, S. L. & Feldmann, R. M. Epibionts on *Dromiopsis* *rugosa* (Decapoda: Brachyura) from the Late Middle Danian limestones at Fakse quary, Denmark: novel preparation techniques yield amazing results. *J. Paleontol.* **78**: 953-960 [https://doi.org/10.1666/0022-3360(2004)078<0953:EODRDB>2.0.CO;2](https://doi.org/10.1666/0022-3360(2004)078%3c0953:EODRDB%3e2.0.CO;2) (2004).
